# Supplementary material for: NLRP3 Inflammasome Is Involved in Cocaine-Mediated Potentiation on Behavioral Changes in CX3CR1-Deficient Mice
Source: J Pers Med. 2021 Sep 27;11(10):963. doi: 10.3390/jpm11100963 (PMC8540128; doi:10.3390/jpm11100963)
Supplement: Supplementary file 1 [file jpm-11-00963-s001.zip › jpm-1333215-supplementary.pdf]

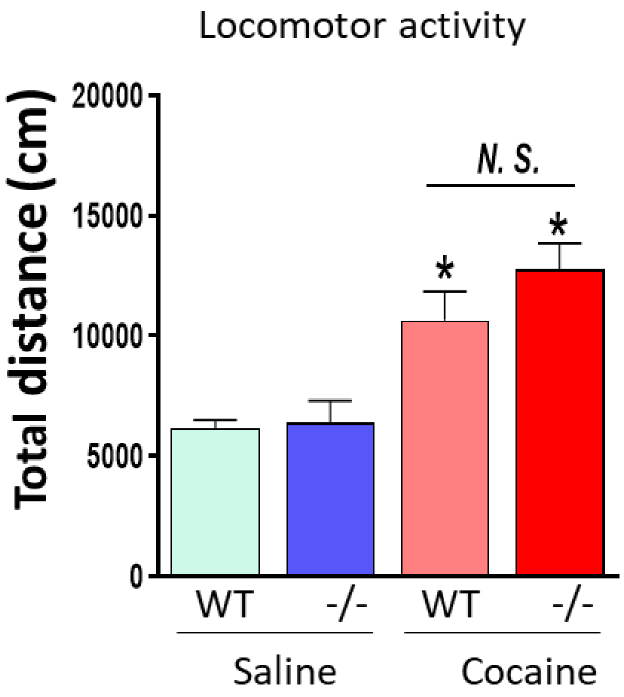

Supplementary Figure S1. One-time cocaine injection did not induce significant difference on the locomotor activity between CX3CR1-/- mice and WT controls (n = 6 - 8; one-way ANOVA analysis. * $P < 0.05$, group of mice with cocaine vs. group of WT with saline. There was no significant difference between WT mice and CX3CR1 -/- mice with one-time injection of cocaine.

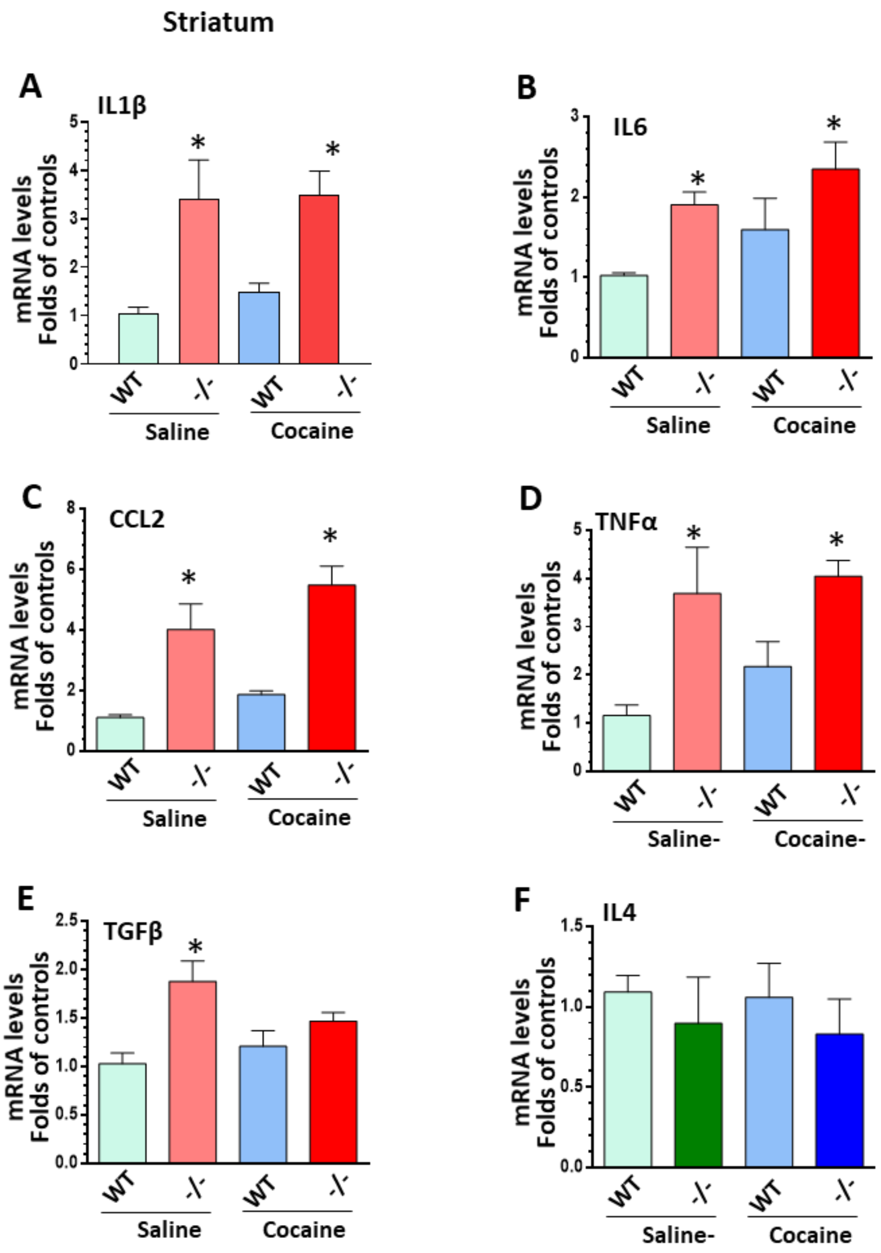

Supplementary Figure S2. The expression levels of pro-inflammatory and anti-inflammatory mediators in the striatum of CX3CR1-/- and WT mice. (**A)** CX3CR1 -/- mice showed increased mRNA levels of IL1β in both cocaine-treated conditions and basal levels (n = 4 - 5, * $P < 0.05$). GAPDH was served as internal controls. (**B**) CX3CR1 -/- mice showed increased mRNA levels of IL6 in both cocaine-treated conditions and basal levels (n = 4 - 5, * $P < 0.05$). GAPDH was served as internal controls. (**C**) CX3CR1 -/- mice showed increased mRNA levels of ccl2 in both cocaine-treated conditions and basal levels (n = 4 - 5, * $P < 0.05$). GAPDH was served as internal controls. (**D**) CX3CR1 -/- mice showed increased mRNA levels of TNFα in both cocaine-treated conditions and basal levels (n = 4 - 5, * $P < 0.05$). GAPDH was served as internal controls. (**E**) CX3CR1 -

/- mice showed increased mRNA levels of TGFβ in basal levels but not in cocaine-treated conditions (n = 4 - 5, * $P < 0.05$). GAPDH was served as internal controls. (**F**) CX3CR1 -/- mice showed no significantly changes on mRNA levels of IL4 in both cocaine-treated conditions and basal levels (n = 4 - 5, * $P > 0.05$). GAPDH was served as internal controls.

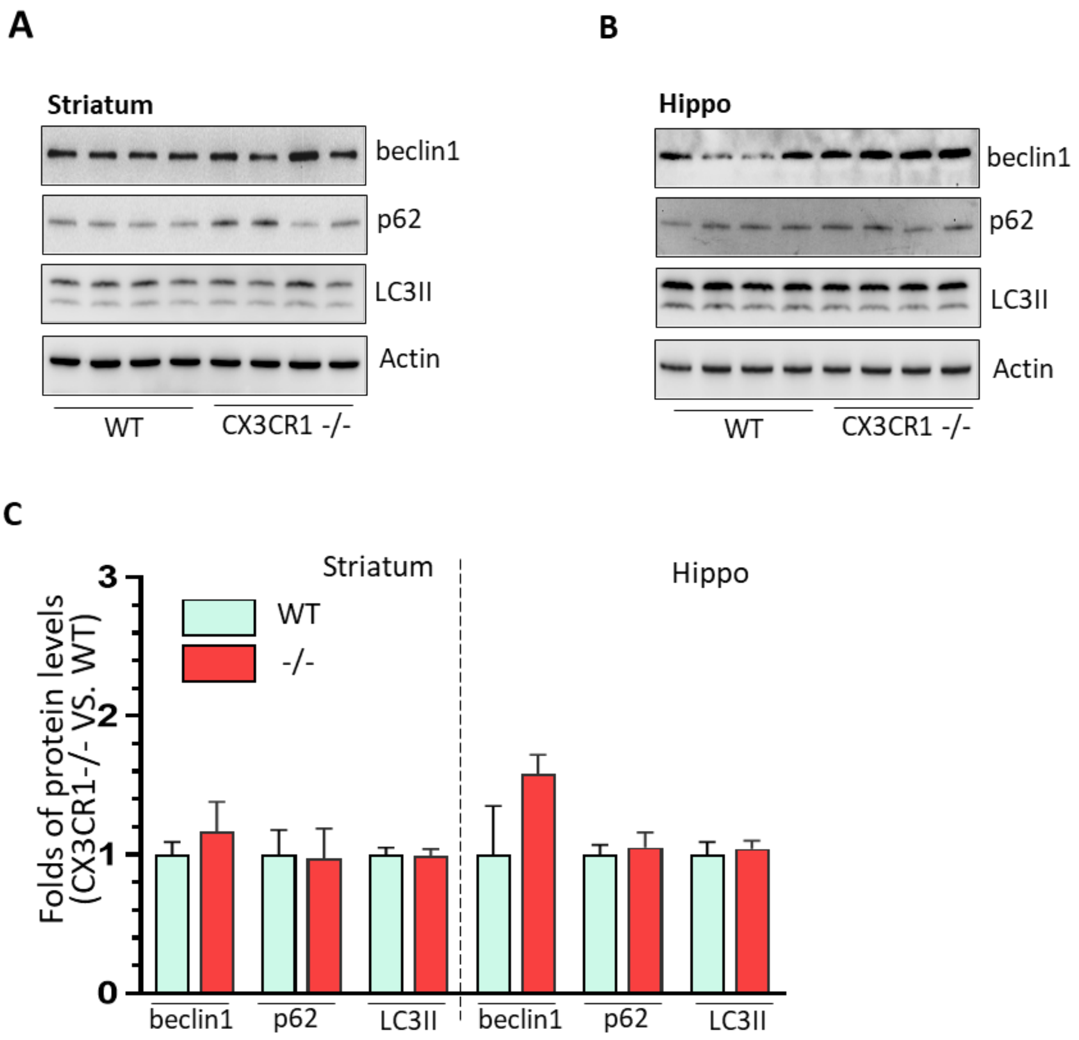

Supplementary Figure S3. The expression levels of autophagic markers in the brains of CX3CR1-/- and WT mice. (**A**) Representative WBs showed no difference on the expression levels of beclin1, p62, and LC3II in the striatum of CX3CR1-/- mice compared to WT mice with cocaine. The levels of β-actin were served as internal controls for equal protein loading ($n = 4–6$, $P > 0.05$). (**B**) Representative WBs showed no difference on the expression levels of beclin1, p62, and LC3II in the hippocampus of CX3CR1-/- mice compared to WT mice with cocaine. The levels of β-actin were served as internal controls for equal protein loading ($n = 4–6$, $P > 0.05$). (**C**) The statistical results for the expression levels of beclin1, p62, and LC3II in the striatum and hippocampus of CX3CR1 -/- and WT mice.

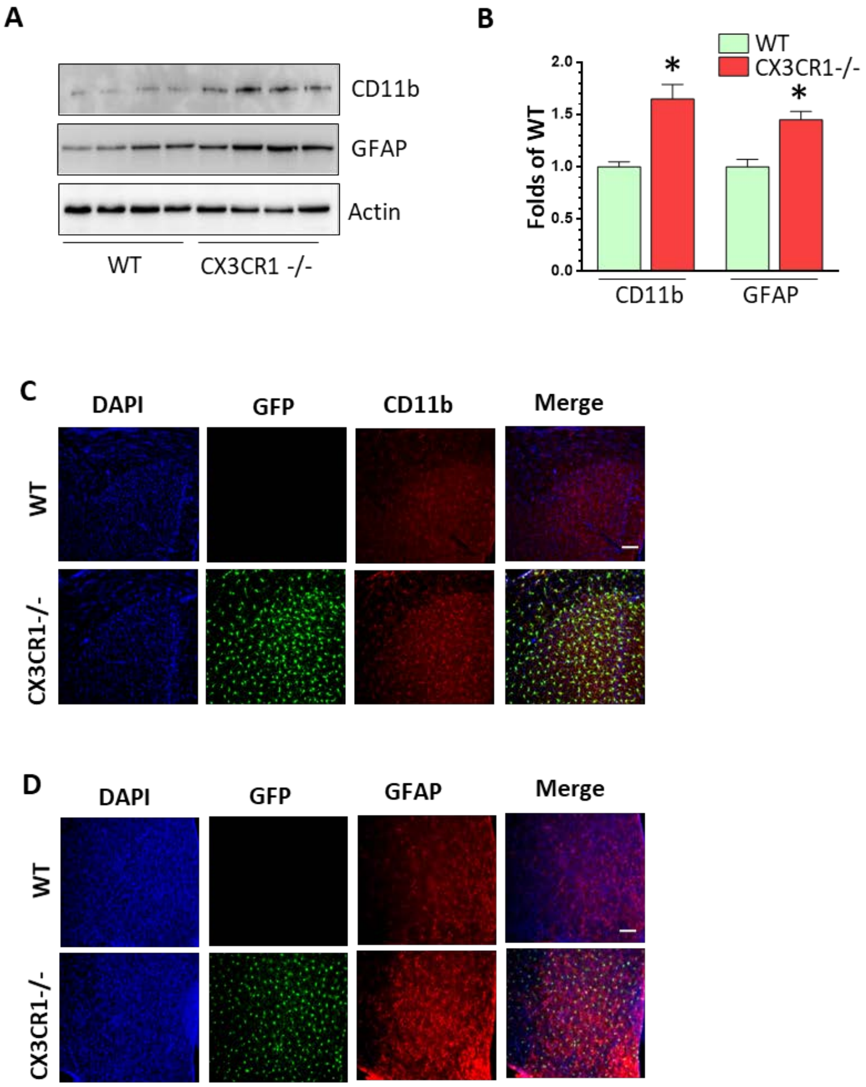

Supplementary Figure S4. CX3CR1 -/- mice showed increased microglia and astrocyte activation levels compared to WT mice under basal conditions. (**A**) Representative WBs showed significant increase on CD11b and GFAP levels in the striatum of CX3CR1 -/- mice compared to WT mice without cocaine administration. The levels of β-actin were served as internal controls for equal protein loading (n = 4 - 6, * P < 0.05). (**B**) Statistic results for WBs shown in (A). (**C**) Immunostaining results showed increased signal intensity of CD11b in CX3CR1 -/- mice compared to WT mice. CD11b was co-localized to GFP signal in CX3CR1 -/- mice. Scale bar: 10 μM. (**D**) Immunostaining results showed increased signal intensity of GFAP in CX3CR1 -/- mice compared to WT mice. GFAP was not co-localized to GFP signal in CX3CR1 -/- mice. Scale bar: 10 μM.

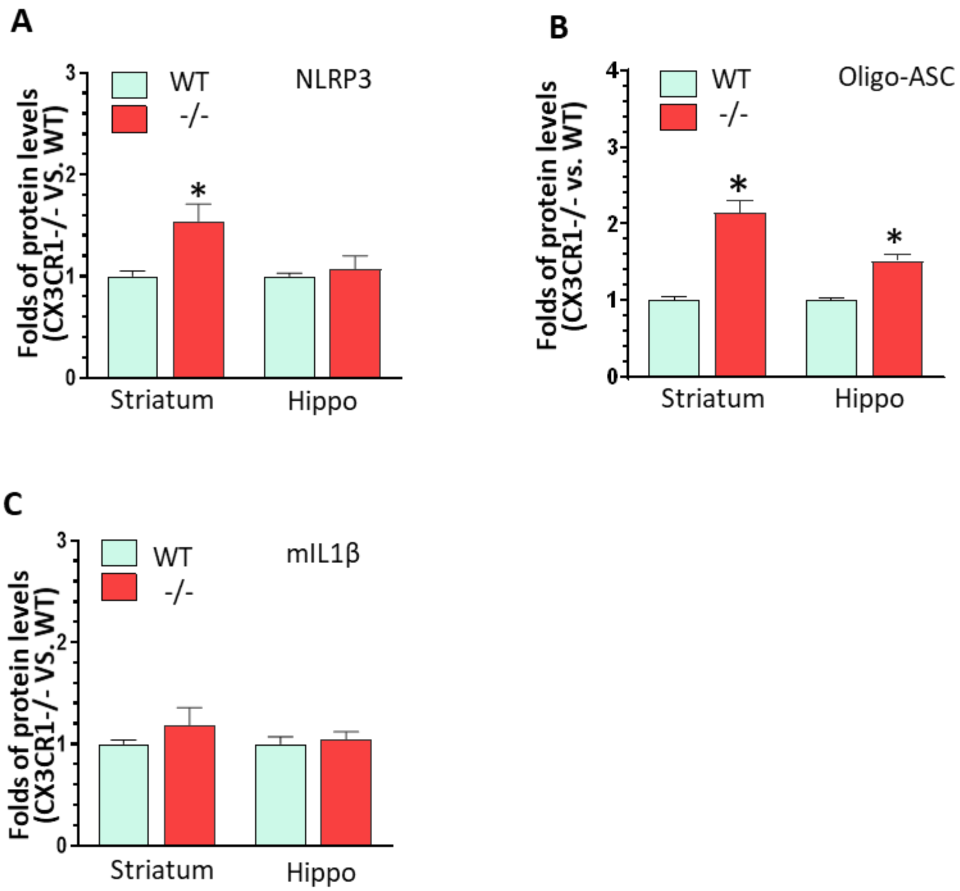

Supplementary Figure S5. CX3CR1 deficiency primed NLRP3 inflammasome in the brain under basal conditions. (**A**) Statistic results showed that CX3CR1 deficiency increased the NLPR3 levels in the striatum under basal conditions (n = 4 - 6, * P < 0.05). (**B**) Statistic results showed that CX3CR1 deficiency increased the ASC oligomerization in the striatum and hippocampus under basal conditions (n = 4 - 6, * P < 0.05). (**C**) Statistic results showed that CX3CR1 deficiency had no impact on the production of mature IL1β in the striaum and hippocampus under basal conditions (n = 4 - 6, P > 0.05).

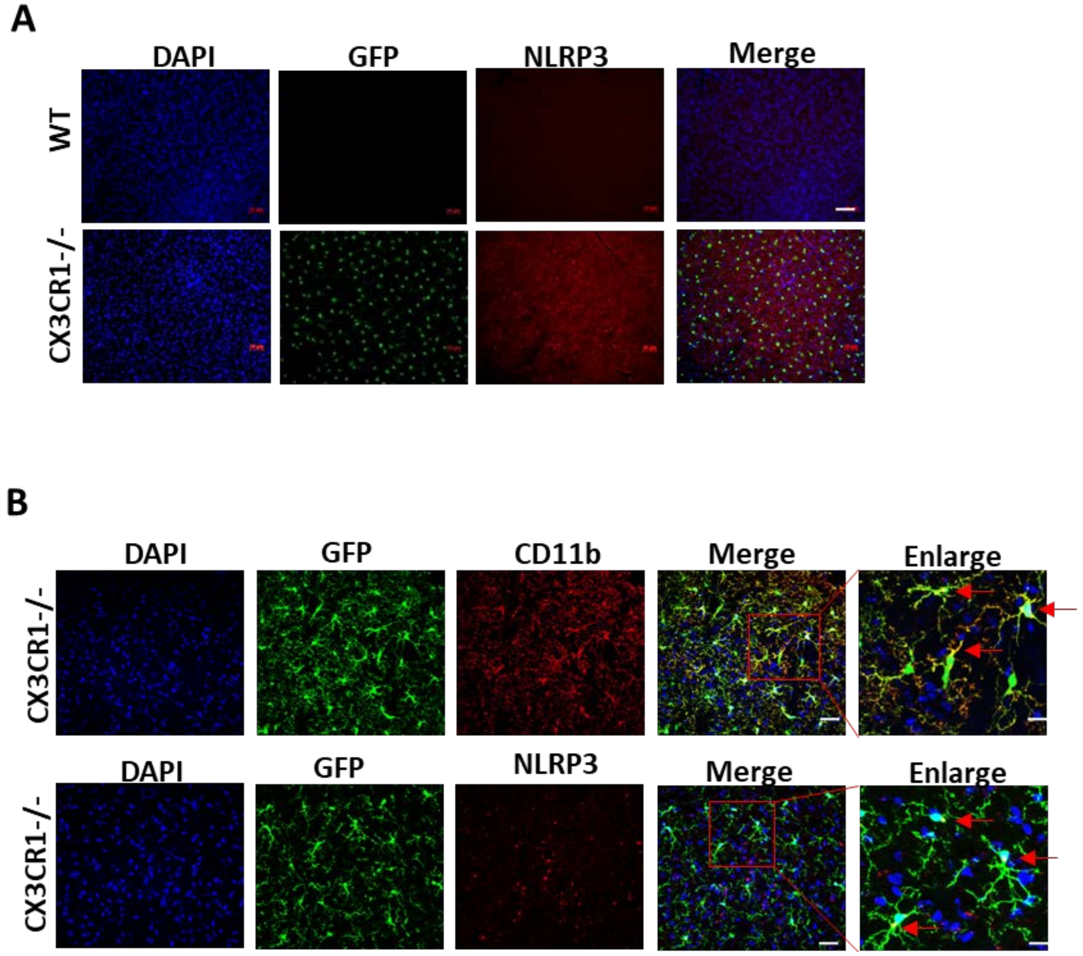

Supplementary Figure S6. CX3CR1 deficiency increased NLRP3 levels in the brain under basal conditions. (**A**) Immunostaining results showed increased signal intensity of NLRP3 in CX3CR1 -/- mice compared to WT mice. Scale bar: 10 μM. (**B**) Enlarged images confirmed that NLRP3 co-localized with GFP in the brains of CX3CR1-/- mice. Scale bar: 2 μM. The co-localization of CD11b and GFP was served as positive controls.

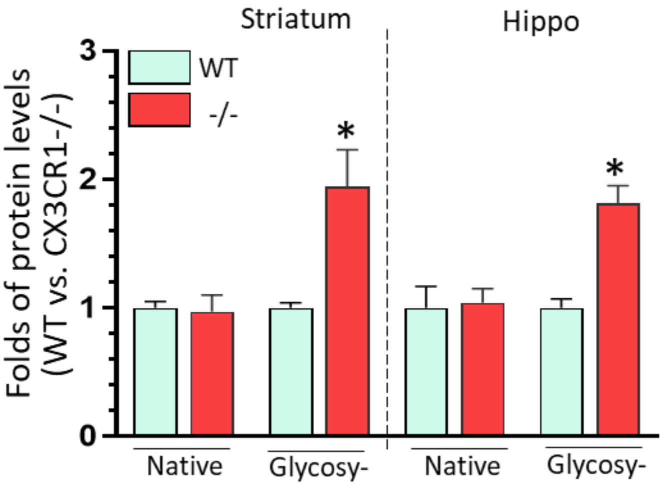

Supplementary Figure S7. CX3CR1 deficiency increased glycosylated LAMP2 levels in the brain under basal conditions (n = 4 - 6, * $P < 0.05$).

Supplementary Table S1. The summary findings of WT and CX3CR1 -/- mice with saline or cocaine injections.

First hit Second hit	**CX3CR1 -/- vs.WT Saline**	**CX3CR1-/-VS.WT cocaine**
**mRNA levels**		
IL1beta<br>IL6<br>TNFa<br>ccl2	up<br>up<br>up<br>up	no change<br>no change<br>no change<br>no change
**behavioral tests**		
locomotor activity<br>CPP	no change<br>no change	up<br>up
**NLRP3 signaling**		
NLRP3 levels<br>oligo ASC<br>mCasp 1<br>glycosylated LAMP2<br>mCat D<br>mILIbeta	up (sriatum)<br>up (both regions)<br>no change<br>up (both regions)<br>up (sriatum)<br>no change	up (both regions)<br>up (both regions)<br>up (both regions)<br>up (both regions)<br>up (both regions)<br>up (both regions)
